# Usage of Silver Nanoparticles in Orthodontic Appliances

**DOI:** 10.3390/ma19010115

**Published:** 2025-12-29

**Authors:** Meigan Niu, Janet Jisoo Lee, Geelsu Hwang, Chun-Hsi Chung, Mark S. Wolff, Zhong Zheng, Chenshuang Li

**Affiliations:** 1 School of Dental Medicine, University of Pennsylvania, Philadelphia, PA 19104, USA; 2 Department of Preventive and Restorative Sciences, School of Dental Medicine, University of Pennsylvania, Philadelphia, PA 19104, USA; 3 Department of Orthodontics, School of Dental Medicine, University of Pennsylvania, Philadelphia, PA 19104, USA; 4 Department of Periodontics, School of Dental Medicine, University of Pennsylvania, Philadelphia, PA 19104, USA

**Keywords:** orthodontics, sliver, nanoparticle, brackets, archwire, elastomeric ligatures, mini-implant, acrylic

## Abstract

Orthodontic treatment, offering significant benefits for oral function and facial aesthetics, is in high demand among both adolescent and adult populations. Orthodontic appliances pose challenges for maintaining oral hygiene and increase the risk of dental and periodontal diseases. With advances in dental materials and the use of nanoparticles, a significant amount of research has focused on modifying orthodontic appliances with nanoparticles to reduce bacterial adhesion and biofilm formation. Silver nanoparticles are one of the most popular antibacterial materials in medical research. This article presents current evidence on silver nanoparticle-incorporated orthodontic appliances, including brackets, molar bands, archwires, elastomeric ligatures, mini-implants, and acrylic retainers. Silver nanoparticles and modified silver nanoparticles exhibit robust antibacterial activity when applied to the surfaces of orthodontic appliances. However, there are exceptions in which, on a few orthodontic appliances, the silver nanoparticle incorporation actually increased biofilm formation. Moreover, a silver nanoparticle incorporation may introduce adverse effects, such as cytotoxicity, and increase surface roughness. It is also worth noting that most of the studies were conducted in vitro. Long-term clinical studies are necessary to evaluate the stability, safety, and clinical efficacy of silver nanoparticle-incorporated orthodontic appliances under real-world conditions.

## 1. Introduction

Orthodontic treatment is a fundamental aspect of modern dentistry, delivering proven benefits in occlusal function, facial aesthetics, and overall oral health. With continued high demand among both adolescent and adult populations, fixed orthodontic appliances, such as brackets, bands, and archwires, remain essential tools for achieving controlled tooth movement and alignment [1]. The specific design that enables effective orthodontic treatment also complicates oral hygiene. Their complex geometries promote food impaction and microbial colonization [2,3,4,5], creating a biofilm formation-favorable environment that is difficult to clean. As a direct result, patients undergoing fixed orthodontic appliance treatment face an increased risk of adverse sequelae, including pronounced plaque accumulation, gingival inflammation, and enamel demineralization [6,7].

In response to these challenges, significant research has focused on modifying orthodontic materials to mitigate bacterial adhesion and biofilm development [8,9]. Nanotechnology, in particular, offers promising strategies for enhancing the antimicrobial properties of orthodontic materials [9,10,11]. Among various nanoparticles, silver nanoparticles (AgNPs), which describe the individual nanoscale silver particles instead of polycrystalline layers in the current review, have garnered considerable attention due to their broad-spectrum antimicrobial activity, stability, and relatively low propensity for inducing microbial resistance [12,13]. Their potent effect is attributed to a high surface area-to-volume ratio that facilitates the release of silver ions (Ag^+^), which exert antimicrobial effects through multiple mechanisms, including the disruption of bacterial membranes, the induction of oxidative stress via reactive oxygen species (ROS), and the inhibition of DNA replication and protein function [14]. 

Currently, AgNP-incorporated orthodontic materials can be categorized into two groups: orthodontic bonding agents and orthodontic appliances. The incorporation of AgNPs into orthodontic bonding agents has demonstrated broad-spectrum activity and the potential to reduce biofilm formation without significantly compromising adhesive performance, as discussed in our previous review [15]. In this manuscript, we will focus specifically on the surface modification of orthodontic appliances, which is particularly important because components such as brackets and archwires constitute the primary surfaces for microbial adhesion and biofilm maturation during orthodontic therapy [16]. The surfaces of these components are ideal for biofilm accumulation, selectively favoring acidogenic and aciduric species like *Streptococcus mutans* [17,18,19]. The specific microbial shift associated with this accumulation is closely linked to the initiation and progression of enamel decalcification and early carious lesions during orthodontic therapy [17,18,19].

Given the persistent challenge of microbial colonization on fixed orthodontic appliances, compounded by the inadequacies of conventional hygiene methods, underscores the need for innovative solutions in orthodontic appliance material. Leveraging the potent antimicrobial properties, AgNPs offer a compelling approach to address this problem, and researchers have explored various surface modification techniques designed to reduce biofilm formation and decrease the risk of enamel demineralization [17,20]. This review appraises and critiques the existing body of evidence on AgNP-integrated orthodontic appliances—encompassing AgNP-incorporated brackets, bands, wires, elastomeric ligatures, retainers, and temporary anchorage devices (TADs). By evaluating their antimicrobial efficacy, biocompatibility, and potential side effects, this synthesis aims to provide insights into further research toward clinically viable and safe antimicrobial applications in orthodontics.

## 2. Silver Nanoparticle-Incorporated Brackets

### 2.1. Antibacterial Effects of AgNP Incorporation

In vitro studies demonstrated that AgNP incorporation, as thin as 4.44 nm, can inhibit the growth of *Staphylococcus aureus* and *Escherichia coli*, while also preventing adhesion of cariogenic bacteria such as *Streptococcus mutans* and *Streptococcus sobrinus* on both stainless steel and ceramic bracket surfaces [21,22] (Table 1). Further studies indicate that the thickness of AgNP incorporation is a factor determining their antibacterial efficacy, where thicker AgNP incorporation on stainless steel brackets provides more potent effects in decreasing *S. mutans* colony counts [23].

Evidence from an in vivo animal model further supports these findings, but the thickness of AgNP incorporation used in the in vivo study is much thicker than that in the in vitro studies. For instance, a study in rats demonstrated that orthodontic brackets coated with a 1-µm layer of AgNP not only reduced *S. mutans* levels in dental plaque but also decreased the incidence of smooth-surface caries [24]. However, the same investigation reported no significant reduction in *S. mutans* colony counts from vestibular smears, nor a decrease in occlusal or sulcal caries [24]. This differential efficacy suggests that AgNPs primarily target bacteria on adjacent, accessible surfaces, with limited effect in more distant anatomic niches. These results highlight the need for further investigation into the sustained antimicrobial behavior of AgNPs within the complex, three-dimensional structure of oral biofilms.

### 2.2. Antibacterial Effect of Modified AgNP-Incorporated Brackets

To enhance antibacterial performance, researchers have explored combining AgNPs with other antimicrobial agents, as listed in Table 1. For instance, a 50 nm dual coating of AgNPs and zinc oxide nanoparticles (AgNPs + ZnONPs) applied to stainless steel brackets (American Orthodontics, 0.018-in. lower premolar) demonstrated significant, sustained inhibition of *S. mutans* and *Lactobacillus acidophilus* growth for up to three months in an in vitro experimental setting [25]. This combinatorial antibacterial effect was superior to that of either nanoparticle type alone. Notably, the antibacterial efficacy did not decline significantly after three months compared with newly coated brackets, suggesting remarkable coating stability and the potential for long-term activity without the need for frequent reapplication or fresh coating preparation [25].

In another approach, chitosan was combined with AgNPs to leverage its polycationic, biocompatible, biodegradable, and bioactive properties [26]. Contrary to the expectation, silver-chitosan nanoparticle (Ag-CsNPs) incorporation exhibited a weaker antibacterial effect than AgNP incorporation alone [26]. Furthermore, the antibacterial performance of both Ag-CsNP and AgNP incorporation also depended on the bracket materials, with coated monocrystalline ceramic brackets exhibited the highest antibacterial activity, followed by coated polycrystalline ceramic and metallic brackets [26]. These findings indicate that the intrinsic properties of the bracket material significantly influence coating performance, underscoring the necessity for material-specific optimization to achieve maximum antimicrobial activity.

**Table 1:** The properties of brackets coated with silver nanoparticles (AgNPs).

Brackets	References	Thickness of AgNP Incorporation	Combinatory Materials	Type of Study	Antibacterial Effect	Side Effects			
						SBS	Toxicity	Surface Roughness	Friction
SS 0.022-in. standard brackets (AO)	Arash et al. 2015 [27]	8–10 µm	N/A	In Vitro	N/A	No change	N/A	Increased	Increased
SS 0.022-in. standard premolar brackets (AO)	Ghasemi et al. 2017 [23]	60 nm and 100 nm	N/A	In Vitro	Thickness-dependent decrease in *S. mutans* colony count	N/A	N/A	Decreased	No significant difference
0.018-in. premolar brackets: InVu Roth (TP Orthodontics), System Alexander LTS (AO), Gemini Roth (3M Unitek), Nu-Edge Roth (TP Orthodontics), Radiance plus Roth (AO)	Jasso-Ruiz et al. 2019 [22]	4.44 nm	N/A	In Vitro	Inhibition of *S. aureus* and *E. coli*	N/A	N/A	N/A	N/A
0.018-in. premolar brackets: InVu Roth (TP Orthodontics), System Alexander LTS (AO), Gemini Roth (3M Unitek), Nu-Edge Roth (TP Orthodontics), Radiance plus Roth (AO)	Jasso-Ruiz et al. 2020 [21]	4.44 nm	N/A	In Vitro	Decreased adhesion of *S. mutans* and *S. sobrinus*	N/A	N/A	N/A	N/A
SS 0.022-in. standard brackets + 0.019 × 0.025-in. SS wires (AO)	Shah et al. 2023 [28]	10 nm	N/A	In Vitro	N/A	N/A	N/A	Decreased in all coated bracket and wire combinations	All coating groups have higher friction than control; coated brackets + uncoated wires showed highest friction.
SS 0.018-in. lower premolar brackets (AO)	Zeidan et al. 2022 [25]	50 nm	ZnONPs	In Vitro	Inhibits *S. mutans* and *L. acidophilus* up to 3 months; AgNPs + ZnONPs has greater effects than AgNPs alone	N/A	N/A	N/A	N/A
0.022-in. upper premolar brackets: Monocrystalline ceramic (Henry Schein Neocrystal Plus Sapphire ceramic), polycrystalline ceramic (3M Neolucent Ceramic Universal), metallic (3M Victory Series Universal)	Tawakal et al. 2023 [26]	N/A	CsNPs	In Vitro	Ag-CsNPs showed weaker antibacterial effects than AgNPs alone; Efficiency varies based on bracket types (monocrystalline ceramic > polycrystalline ceramic > metallic)	N/A	N/A	AgNPs and Ag-CsNPs decreased surface roughness of monocrystalline ceramic brackets, increased surface roughness of polycrystalline ceramic brackets; AgNPs and Ag-CsNPs had a non-significant effect on surface roughness of metallic brackets	AgNPs and Ag-CsNPs incorporation decreased friction of mono and polycrystalline ceramic brackets and increased friction of metallic brackets
Gemini Roth mandibular incisor brackets (3M Unitek)	Metin-Gürsoy et al. 2017 [24]	1 µm	N/A	In Vivo (Wister rats)	Reduced *S. mutans* in dental plaque but not in vestibular smear; Reduced smooth surface caries but not occlusal or sulcal caries.	N/A	No behavioral or weight change, no mortality	N/A	N/A

SS: stainless steel; SBS: shear bonding strength; ZnONPs: Zinc oxide nanoparticles; Ag-CsNPs: Silver chitosan nanoparticles; N/A: not reported in the primary literature.

### 2.3. Side Effects

#### 2.3.1. Surface Roughness

Studies examining the impact of different thicknesses of AgNP incorporation on stainless steel standard orthodontic brackets have yielded mixed results as listed in Table 1. While thinner coatings of 10 nm, 60 nm, and 100 nm reduced surface roughness [23,29], thicker coatings of 8–10 µm increased roughness [27]. These conflicting results indicate a threshold: nanoscale AgNP incorporation may enhance the smoothness of stainless-steel brackets, but microscale coatings can adversely affect their surface topography.

The effect of AgNP-based incorporation on bracket surface roughness is also highly dependent on the substrate material of the brackets. A comparative study demonstrated that while both AgNP and Ag-CsNP incorporation reduced surface roughness of monocrystalline ceramic brackets, they increased the roughness of polycrystalline ceramic brackets and had no significant effect on metallic brackets [26]. Although the specific coating thicknesses were not reported, these divergent results indicate that the intrinsic properties of the bracket material may govern the resulting surface characteristics. This dependency highlights the importance of tailoring coating parameters to specific bracket compositions to achieve the desired clinical performance.

#### 2.3.2. Friction

Most studies report increased frictional forces of metallic brackets post AgNP or Ag-CsNP incorporation [26,27,28,29], though one investigation found no significant change despite measurable reductions in surface roughness [23], as listed in Table 1. On the other hand, on both mono- and polycrystalline ceramic brackets, both AgNP and Ag-CsNP incorporation produced a slight, non-significant reduction in friction [26]. These divergent findings suggest that surface roughness alone may not be the sole determinant of friction at the bracket-wire interface, indicating the involvement of other factors, such as surface chemistry, coating adhesion, and archwire characteristics.

#### 2.3.3. Other Side Effects

Current available evidence on other side effects of AgNP-incorporated brackets is limited. One study investigated the shear bonding strength (SBS) of stainless-steel brackets and found no significant change after AgNP incorporation [27]. An in vivo study in rats reported no systemic adverse effects, such as behavioral changes, weight loss or gain, or mortality, following exposure to AgNP-incorporated brackets [24], indicating a favorable preliminary biocompatibility profile.

### 2.4. Summary

Overall, current evidence substantiates the antibacterial potential of AgNP-incorporated orthodontic brackets, with in vitro and in vivo studies confirming reductions in bacterial adhesion or colony count, and in incidence of early smooth surface caries. The efficacy and physical properties of these coatings, however, are highly dependent on several factors, including but not limited to coating thickness, chemical composition (e.g., combinations with other materials), and the substrate bracket materials. A possible limitation is that the antibacterial action appears confined to superficial biofilm layers, underscoring the need for strategies that enhance biofilm penetration. Future research should optimize AgNP formulations for specific bracket substrates, evaluate long-term clinical performance and stability, and access the critical balance between potent antibacterial activity and minimal adverse effects on surface roughness, friction, bond strength, and overall biocompatibility.

## 3. Silver Nanoparticle-Incorporated Bands

Although the literature on AgNP-incorporated bands is less extensive than that of coated brackets, existing in vitro studies confirm their antimicrobial potency. For example, studies demonstrated that coating stainless steel bands with AgNPs effectively reduced bacterial counts and inhibited plaque accumulation [30,31] (Table 2). Additionally, AgNP incorporation exhibited promising biocompatibility, with cell viability assays showing minimal cytotoxicity in both L929 mouse fibroblasts (>80% viability) and human gingival fibroblasts (85.7% viability) [30,31]. These findings collectively highlight the potential of AgNP-incorporated bands to impart antimicrobial activities while maintaining a favorable biocompatibility profile. However, the current evidence base is constrained by its reliance on short-term in vitro models. To validate these promising results, further investigations should prioritize long-term clinical studies that assess the coatings’ durability, stability, and overall safety in patients’ oral environment.

## 4. Silver Nanoparticle-Incorporated Archwires

As brackets and bands are cemented onto tooth surfaces for extended periods, assessing the long-term in vivo antibacterial performance of their AgNP incorporation remains a challenge. Consequently, a strategic shift toward components that can be easily changed during each orthodontic visit, such as orthodontic archwires and ligatures, was promoted, enabling AgNP incorporation to be regularly replenished to ensure consistent antimicrobial activity. 

### 4.1. Antibacterial Effects of AgNP-Incorporated Archwires

Multiple studies confirmed AgNP incorporation on stainless steel (SS), nickel-titanium (NiTi), and copper-nickel-titanium (CuNiTi) archwires effectively inhibit *S. mutans* growth, reduce adhesion of *S. mutans* and *S. aureus*, and suppress biofilm formation [17,32,33] (Table 3). Their antibacterial efficacy, however, is influenced by several variables, including nanoparticle size, bacterial serotype, and archwire composition. For instance, Espinosa-Cristóbal et al. reported that smaller-diameter AgNPs exhibited superior efficacy against *S. mutans* growth across all three tested archwire types [32]. Meanwhile, Nafarrate-Valdez et al. observed that the coating provided greater inhibition against *S. mutans* serotype c than against serotype k, with coated CuNiTi archwires exhibiting the most substantial anti-adherent effects, followed by coated NiTi and SS archwires [17].

It is worth noting that brand-specific variability further complicates the clinical translation of AgNP-incorporated archwires. For example, Gonçalves et al. reported a significant improvement in antibacterial activity with AgNP-incorporated Abzil^®^ SS wires, but not with AgNP-incorporated Orthometric^®^ counterparts [33]. While the specific alloy compositions were undisclosed by the manufacturers, these divergent outcomes strongly suggested that subtle differences in the elemental composition of the wires critically influence the coating’s antibacterial performance, warranting further investigation to explicitly account for and examine these material-based variables to ensure predictable and effective antimicrobial outcomes. 

### 4.2. Antibacterial Effect of Modified AgNP-Incorporated Archwires

To enhance the antibacterial performance of AgNP-incorporated archwires, researchers have explored the synthesis of nanoparticles using plant extracts, as listed in Table 3. For example, AgNPs synthesized from adding AgNO_3_ to vanillin (an active component of Vanilla Planiflora plant extract) demonstrated profound activity against *S. mutans* when coating onto the surface of CuNiTi wires [34]. Meanwhile, Anand et al. combined plant-derived AgNPs—synthesized by adding AgNO_3_ to extract of *Ocimum sanctum*, *Ocimum tenuiflorum*, *Solanum surattense*, or *Syzgium aromaticum*—with titanium dioxide nanoparticles (TiO_2_NPs) [35]. The antibacterial activity was potent and relied on the source of the AgNPs: AgNPs derived from *S. surattense* exhibited broad-spectrum antibacterial activity against *B. subtilis, Enterobacter, Flavobacterium, P. aerogenosa* (SP1&SP2)*,* and *E. coli,* while those from *O. sanctum* and *O. tenuiflorum* acted more selectively against *P. aerogenosa* (SP3) and *S. albus*, respectively [35]. These findings suggest that the phytochemicals may determine critical physicochemical properties—such as size, shape, and surface chemistry—of the AgNPs, which, in turn, govern the spectrum and specificity of antimicrobial action, as well as their composite partners.

Other composite formulations have also demonstrated potential. For instance, as summarized in Table 3, chitosan-silver nanocomposite (CS-Ag) incorporation has demonstrated superior inhibition of both Gram-positive and Gram-negative bacteria compared to polyvinyl alcohol-silver nanocomposites (PVA-Ag) [36]. A direct comparison with pure AgNP incorporation, however, was not performed in this study. Thus, it remains unclear if CS-Ag composite offers a more potent antibacterial effect over AgNPs alone, presenting a gap for future comparative investigation.

### 4.3. Side Effects

#### 4.3.1. Physical-Chemical Property

The chemical and physical characteristics of AgNPs influence their stability and antibacterial performance. Gonçalves et al. demonstrated that hydrothermal synthesis enabled successful coating of AgNPs on SS archwires without significantly altering the elemental composition, suggesting a feasible approach for preserving both structural and antibacterial integrity [33]. Meanwhile, Cristobal et al. noted that, compared with larger counterparts, smaller AgNPs have lower charge and are thus more prone to agglomeration, which can compromise their long-term stability and antimicrobial performance [32].

#### 4.3.2. Surface Roughness

The impact of AgNP incorporation on surface roughness—a key factor influencing both sliding mechanics and bacterial adhesion—varies considerably across studies, as listed in Table 3. For instance, Choi et al. reported a significant increase in surface roughness on AgNP-incorporated NiTi archwires, which may compromise sliding efficiency and promote bacterial attachment [37]. Conversely, Abdallah et al. observed that NiTi archwires coated with PVA-Ag or CS-Ag nanocomposites exhibited an overall smoother surface, with CS-Ag incorporation providing superior minimization of surface irregularities [36]. Similarly, CuNiTi wires coated with vanillin-mediated AgNPs also demonstrated a decrease in surface roughness [34]. These divergent findings highlight that the resultant surface topography is highly dependent on the specific archwire substrate, coating composition, and application technique.

#### 4.3.3. Friction

Friction at the bracket-archwire interface is a critical determinant of orthodontic tooth movement efficacy [29]. One study found that 10 nm AgNP incorporation did not considerably alter the friction of small-sized (0.017 × 0.025-in.) SS archwires but did significantly reduce the friction of larger wires (0.019 × 0.025-in.) [29], suggesting a wire-size-dependence effect. Interestingly, Abdallah et al. found that both CS-Ag and PVA-Ag incorporation reduced the surface roughness of 0.016 × 0.022-in. NiTi archwires and stated that the friction coefficient was directly proportional to surface roughness [36]. The discrepancies between these two studies may be attributed to differences in the substance material and size of archwire, as well as the type of AgNPs. However, future systematic investigations are warranted to delineate the specific influence of each variable on the frictional behavior of AgNP-incorporated archwires.

**Table 3:** The properties of archwires coated with silver nanoparticles (AgNPs).

Archwires	References	Combinatory Materials	Type of Study	Antibacterial Effect	Side Effect			
					Physical-Chemical Property	Surface Roughness	Friction	Cytotoxicity
0.014-in. Biopolymer-coated esthetic NiTi	Choi et al. 2015 [37]	N/A	In Vitro	N/A	N/A	Increased	N/A	N/A
NiTi, CuNiTi, and SS (AhKimPech^TM^)	Espinosa-Cristóbal et al. 2018 [32]	N/A	In Vitro	growth inhibition of *S. mutans* on all tested wires, and more effective with smaller AgNPs	electrical charges: larger AgNP > smaller AgNP	N/A	N/A	N/A
NiTi, CuNiTi, and SS (AhKimPech^TM^)	Nafarrate-Valdez et al. 2022 [17]	N/A	In Vitro	anti-adherent effect: CuNiTi > NiTi > SS	N/A	N/A	N/A	N/A
0.016 × 0.022-in. NiTi (OrthoPro, FL, USA)	Abdallah et al. 2024 [36]	CS-Ag and PVA-Ag	In Vitro	Antimicrobial Gram+ and Gram-: CS-Ag > PVA-Ag at highest concentrations of AgNPs for both	N/A	Significant decrease among all AgNP and nanocomposite incorporation Surface irregularities: PVA-Ag > CS-Ag	Friction coefficient directly proportional to surface roughness	N/A
0.017 × 0.025-in. SS, 0.019 × 0.025-in. SS	Shah et al. 2018 [29]	N/A	In Vitro	N/A	N/A	N/A	0.017 × 0.025-in.: no effect; 0.019 × 0.025-in.: reduced.	N/A
SS (Abzil® & Orthometric®)	Gonçalves et al. 2020 [33]	N/A	In Vitro	Abzil®: significant reduction in *S. mutans* and *S. aureus* biofilm formation; Orthometric®: no significant change	no effect	N/A	N/A	N/A
CuNiTi	Anishya et al. 2024 [34]	Vanillin plant extract	In Vitro	antibacterial action against *S. mutans*	N/A	Decreased	N/A	12.5 and 75 µg/mL: 90% viability of HGF; 100 µg/mL: 80% viability of HGF.
Austenitic SS	Anand et al. 2023 [35]	TiO_2_NPs Plant extract	In Vitro	AgNPs synthesized from *Solanum surattense*, *Ocimum sanctum*, *Ocimum tenuiflorum*, and *Syzygium Aromaticum* showed various antimicrobial activities	N/A	N/A	N/A	N/A

SS: stainless steel, NiTi: nickel-titanium, CuNiTi: copper-nickel-titanium, CS: chitosan, PVA: polyvinyl alcohol, HGF: human gingival fibroblasts. N/A: not reported in the primary literature.

#### 4.3.4. Cytotoxicity

The only available investigation into the cytotoxicity of AgNP-incorporated archwires involved vanillin-mediated AgNPs, reporting a concentration-dependent decrease in human gingival fibroblast viability; however, cell viability remained above 80% across all tested concentrations [34], indicating an overall low cytotoxicity.

### 4.4. Summary

The current evidence suggests that AgNP-incorporated archwires exhibit substantial antibacterial potency, contingent upon several variables, including wire material composition, AgNP dimensions, and the target bacterial serotype. Emerging strategies employ plant-mediated synthesis and composite nanoparticles may further enhance their antibacterial performance and target specificity.

It is worth noting that the clinical potential of AgNP-incorporated archwires is hindered by two primary obstacles: the long-term stability of AgNPs and their variable effects on surface properties, which are critical to orthodontic treatment efficiency. Consequently, further research is needed to optimize coating methodologies, validate mechanical performance, and confirm long-term safety and effectiveness.

## 5. Silver Nanoparticle Incorporated-Elastomeric Ligatures

Despite their utility in archwire engagement, elastomeric ligatures are highly susceptible to plaque accumulation and bacterial colonization [38]. To mitigate this drawback, AgNPs have been integrated into the ligature materials. Furthermore, the routine replacement of ligatures at adjustment appointments facilitates the consistent reapplication of fresh AgNP incorporation, maintaining their antimicrobial efficacy throughout treatment.

### 5.1. Antibacterial Effect

In vitro analyses confirm that AgNP-incorporated elastomeric ligatures exhibit significant antibacterial activity against common oral pathogens, including *S. mutans*, *Lactobacillus casei*, *S. aureus*, and *E. coli* [39,40,41] (Table 4). However, these promising laboratory results have not been consistently translated to the clinical setting. For example, a human in vivo trial found no significant reduction in *S. mutans* levels when comparing AgNP-incorporated ligatures to their controls [38]. Thus, a critical gap exists between in vitro performance and clinical effectiveness, underscoring the need for further rigorous clinical validation.

To enhance the antibacterial and anti-demineralization properties of AgNP-incorporated elastomeric ligatures, Chio et al. developed a composite coating of nanosilver fluoride (NSF)-ethyl cellulose (EC). This modification was designed to provide a sustained co-release of Ag and fluoride [42,43]. In vitro evaluations showed that these modified ligatures significantly inhibited *S. mutans* growth, decreased overall biofilm thickness, and diminished enamel demineralization [42,43]. However, the clinical translation of these promising results requires confirmation through in vivo studies.

### 5.2. Side Effects

Compared to controls, AgNP-incorporated elastomeric ligatures generally exhibit favorable physical characteristics, including increased strength, tension, and displacement [39], and surface-free energy [40], with reduced surface roughness [40], as summarized in Table 4. Noteworthily, these benefits must be balanced against potential limitations in long-term durability. For instance, Pasala et al. observed a gradual release of AgNPs into artificial saliva, accompanied by decreased durability over time, indicating material degradation [41]. However, another study found that NSF-EC incorporation had no significant effect on the tensile force of the elastomeric ligatures [42], suggesting that specific incorporation formulations can mitigate adverse effects on mechanical properties. 

### 5.3. Summary

In summary, while AgNP-incorporated elastomeric ligatures exhibit promising in vitro antibacterial activity and favorable physical characteristics, such as increased strength and reduced surface roughness, their clinical translation remains unproven. An initial in vivo study failed to demonstrate a significant reduction in bacterial colonization, and concerns regarding long-term durability and nanoparticle leaching into saliva persist. Therefore, further research should prioritize well-designed clinical studies to validate antimicrobial efficacy, clarify the role of material combinations, and evaluate long-term biocompatibility and mechanical stability in the oral environment.

## 6. Silver Nanoparticle-Incorporated Orthodontic Mini-Implants

Orthodontic temporary anchorage devices (TADs), also referred to as mini-implants or miniscrews, are widely used in modern orthodontics to provide additional anchorage and support for tooth movement and maxillary skeletal expansion. Their clinical failure, however, can delay or necessitate changes to the orthodontic treatment plan, prolong treatment time, and result in ineffective or suboptimal outcomes. Common causes of TAD failure include peri-implantitis and low bone quality/density. Given that silver incorporations have been shown to mitigate infection and enhance osseointegration around orthopedic hardwires and dental implants [44,45,46,47], clinicians and researchers have sought to extend these beneficial effects to orthodontic mini-implants by AgNP incorporation (Table 5).

### 6.1. Antibacterial Effect

Evidence regarding the antibacterial properties of AgNP-incorporated TADs remains inconclusive. As listed in Table 5, while Andini et al. reported that AgNP-incorporated TADs prevented *P. gingivalis* colonization as effectively as autoclave sterilization [48], Venugopal et al. [49] and Sycińska-Dziarnowska et al. [50] observed no antibacterial activity of AgNP-incorporated TADs. These discrepancies underscore the need for further investigations to clarify the parameters governing their antibacterial performance.

To enhance their efficacy, AgNPs have been combined with other bioactive coatings. For instance, a composite coating of hydroxyapatite (HA) and chitosan biopolymer with AgNPs (BP-AgNP) on Ti6a14V mini-implants created clear inhibition zones against *S. mutans, S. sanguinis, and Aggregatibacter actinomycetemcometans,* an effect not observed with sole AgNP incorporation [49]. Similarly, combining HA with AgNPs conferred broad-spectrum in vitro antimicrobial resistance against pathogens such as *S. mutans, S. aureus, E. faecalis, E. aeruginosa, E. coli*, and *C. albicans* [51]. More importantly, this composite also demonstrated significant reductions in total oral bacterial count in an in vivo rabbit model [52], validating its potential for clinical application. Conversely, coatings combining calcium and phosphorus—two bioactive elements known to improve microbial resistance—with AgNPs paradoxically resulted in a statistically significant increase in bacterial biofilm on TADs compared with control implants [50].

### 6.2. Osseointegration

Osseointegration, defined as the “direct anchorage of an implant by formation of bony tissue around the implant without the growth of fibrous tissue at the bone-implant interface,” is a critical determinant of mini-implant success [52]. In vivo studies in rabbit models have shown that coating with HA-AgNP increases bone density at the implant interface, suggesting that this combinatorial incorporation can improve implant stability by promoting bone formation [52].

### 6.3. Side Effects

#### 6.3.1. Cytotoxicity

Despite their antimicrobial benefits, some composite coatings raise concerns about cytotoxicity. For example, HA-AgNP-incorporated mini-implants demonstrated reduced cytocompatibility compared with uncoated and ZnO-incorporated controls [51]. Cell viability assays revealed notable toxicity, particularly in bone-forming and connective tissue cells: viability fell below 60% in osteoblasts, osteocytes, and fibroblasts [51].

#### 6.3.2. Surface Roughness

The surface topography of mini-implants presents a critical design challenge: while increased roughness enhances osseointegration by improving mechanical interlock with bone, it can also promote bacterial colonization and biofilm formation [50]. Therefore, an optimal surface must strike a balance between these competing requirements. In this context, the impact of AgNP incorporation on surface roughness varies. Sole AgNP incorporation has been shown to reduce roughness, likely due to their lower porosity, whereas composite coatings, such as using AgNP + calcium phosphate (CaP), significantly increased roughness compared to uncoated controls [50,53]. 

### 6.4. Summary

In summary, the efficacy of AgNP-based incorporation on mini-implants is highly formulation-dependent. Composite coatings, such as BP-AgNPs and HA-AgNPs, demonstrate enhanced, broad-spectrum activity compared to AgNPs alone. Nevertheless, key limitations persist, such as the cytotoxicity of HA-AgNP incorporation and the counterproductive increase in biofilm formation on rougher AgNP + CaP-incorporated surfaces. For clinical translation, future studies are warranted to develop advanced coatings that harmonize antimicrobial performance with osteogenic potential and minimal cellular toxicity, ultimately requiring rigorous clinical validation.

**Table 5:** The properties of orthodontic mini-implants coated with silver nanoparticles (AgNPs).

Orthodontic Micro-Implant	Reference	Combinatory Material	Type of Study	Antibacterial Effect	Cytotoxicity	Osteointegration	Surface Roughness
7 mm length x 1.2 mm diameter TADs	Andini et al. 2019 [48]	N/A	In Vitro	Bactericidal effect in *P. gingivalis*	N/A	N/A	N/A
Dentos® Ti6Al4V (Dalseo-Gu, Daegu, Republic of Korea)	Venugopal et al. 2017 [49]	BP	In Vitro	AgNP: no effects; BP-AgNP: growth inhibition of *S. mutans, S. sanguinis,* and *A. actinomycetemcometans*	N/A	N/A	N/A
Tomas® TMS system Titanium mini-screws (Dentaurum, Germany)	Abo-Elmahasen et al. 2023 [51]	HA	In Vitro	Antibacterial and antifungal effect against *S.mutans, S.aureus, E.faecalis, E.aeruginosa, E.coli, C.albicans*	Less cytocompatibility than ZnO on oral epithelial cells, osteoblasts/osteocytes, and human fibroblasts: Cell viability <100%, <60%, <60%; Toxicity <20%, <55%, <60%, respectively	N/A	N/A
Tomas® TMS system Titanium mini-screws (Dentaurum, Germany)	Abo-Elmahasen et al. 2023 [52]	HA	In Vivo (*Albino rabbits*)	Significant decrease in total bacterial count of the oral cavity	N/A	Increased bone density	N/A
Dentos® Ti6Al4V (Dalseo-Gu, Daegu, Republic of Korea)	Sycińska-Dziarnowska et al. 2024 [53]	CaP	In Vitro	N/A	N/A	N/A	AgNP+CaP > control > AgNP
Dentos® Ti6Al4V (Dalseo-Gu, Daegu, Republic of Korea)	Sycińska-Dziarnowska et al. 2025 [50]	CaP	In Vitro	*S. aureus, E. coli*, and *S. mutans* biofilm formation: AgNP + CaP > AgNP = control	N/A	N/A	AgNP+CaP > control > AgNP

BP: biopolymer, HA: hydroxyapatite, CaP: Calcium Phosphorus, Ti6Al4V: titanium alloy; N/A: not reported in the primary literature.

## 7. AgNPs Incorporation in Orthodontic Retainers

While ensuring a proper fit and enforcing a rigorous regimen of cleaning are always imperative for retainer longevity and oral tissue health, this section focuses on how AgNP incorporation addresses these challenges within polymethyl methacrylate (PMMA) retainers, since PMMA is the most used material for fabricating orthodontic retainers and removable appliances, valued for its durability and moldability [54].

### 7.1. Antibacterial Effect

Human clinical trials have evaluated PMMA retainers incorporating AgNPs via either direct (NanoAg-I-PMMA) or in situ (NanoAg-IS-PMMA) (Table 6). Both AgNP-incorporated retainers significantly suppressed biofilm formation and inhibited the growth of S. mutans, S. sobrinus, L. acidophilus, and L. casei at 0.5% (w/w) and 500 ppm concentrations over four and seven weeks, respectively [55,56]. Notably, the NanoAg-IS-PPMA formulation demonstrated greater antibacterial performance, attributed to its superior nanoparticle dispersion [55]. Given that PMMA retainers are often worn for extended periods, it is crucial for them to have a long-term antimicrobial activity. However, an in vitro study conducted by Williams et al. suggests this may be challenging to achieve. They found that while PMMA incorporated with 0.4% (w/w) Ag benzoate effectively resisted the formation of *S. mutans* biofilm after a short-term washout, it lost its antimicrobial properties following a prolonged six-month washout [57], indicating that the sustained antimicrobial efficacy of silver-loaded PMMA may be limited.

### 7.2. Side Effects

At lower concentrations, AgNP incorporation appears to be safe and esthetically acceptable. For instance, 500 ppm AgNP incorporation was associated with no cytotoxicity or discoloration in the clinical tests [56]. Similarly, at 0.4% (w/w), no statistically significant differences in surface roughness of PMMA retainers were attributed to AgNP-incorporation, despite a slightly more corrugated surface texture [57]. 

The incorporation of AgNPs at higher concentrations, however, can negatively affect the mechanical properties of orthodontic retainers. For example, 5% (w/w) AgNPs significantly decreased the tensile strength of the incorporated PMMA retainers, likely due to nanoparticle aggregation and consequent reduction in effective density of AgNPs [58]. Given that PMMA already has inherent limited mechanical resilience, the careful optimization of incorporated AgNP concentration is critical to prevent further compromising the retainers’ structural integrity.

### 7.3. Summary

The incorporation of AgNPs into PMMA orthodontic retainers offers short-term antibacterial benefits, good biocompatibility, and minimal esthetic compromise at lower concentrations. However, the clinical promise of this type of modified retainer is tempered by concerns about the durability of antimicrobial efficacy, especially due to washout, and the risk of mechanical integrity loss with high-concentration AgNPs incorporation. To address these concerns, future work is needed to optimize coating formulations to achieve sustained antimicrobial potency while preserving the retainers’ mechanical and esthetic integrity.

## 8. Conclusions

AgNPs demonstrate considerable potential as a broad-spectrum antimicrobial agent for a wide array of orthodontic appliances, including brackets, bands, archwires, elastomeric ligatures, temporary anchorage devices, and PMMA retainers. The development of composite or hybrid formulations incorporating materials such as ZnO, HA, CaP, or chitosan further enhances their antibacterial and antifungal efficacy.

Despite these advantages, the clinical translation of AgNP technology is not without its challenges. The incorporation of AgNPs can critically alter the key characteristics of the substance materials, such as surface roughness, frictional properties, mechanical strength, and biocompatibility, with these effects being highly dependent on AgNPs’ particle size and concentration, as well as the type of substrate material. Furthermore, while in vitro studies consistently report significant antimicrobial benefits, robust in vivo and clinical evidence remains limited. This gap underscores the necessity for further research to fully elucidate the risks and benefits of AgNP usage in orthodontics, particularly regarding long-term stability, safety, and effectiveness under real-world conditions. 

Overall, AgNP incorporation represents a promising avenue for mitigating microbial complications in orthodontic treatment, such as white spot lesions and gingival inflammation. Future efforts focused on refining formulations and conducting rigorous clinical trials will be essential to translate this promise into safe, effective, and clinically viable solutions that may significantly improve patient outcomes while maintaining the structural and functional integrity of orthodontic materials. By sharing our thoughts here, we aim to inspire enthusiasm for conducting clinical validation to bridge the translational gap between AgNPs’ experimental potential and their clinically demonstrated outcomes. We will also continue to follow up on clinical evaluations to validate our conclusions.

## Figures and Tables

**Table 2:** The properties of orthodontic bands coated with silver nanoparticles (AgNPs).

Bands	References	Combinatory Materials	Type of Study	Antibacterial Effect	Side Effect			
					Toxicity	Surface Roughness	Friction	Discoloration
Stainless steel bands (Desires)	Prabha et al. 2016 [30]	N/A	In Vitro	antibacterial effect against Gram^+^	>80% L929 viability	N/A	N/A	N/A
Stainless steel orthodontic bands (AO)	Bahrami et al. 2023 [31]	N/A	In Vitro	Reduce *L. acidophilus*, *C. albicans*, and *S. mutans*	85.7% HGF viability	N/A	N/A	N/A

L929: mouse fibroblast cells (National Centre for Cell Sciences, Pune); HGF: human gingival fibroblasts; N/A: not reported in the primary literature.

**Table 4:** The properties of elastomeric ligatures coated with silver nanoparticles (AgNPs).

Elastomeric Ligatures	Reference	Combinatory Materials	Type of Study	Antibacterial Effect	Side Effects		
					Physical Property	Surface Roughness	Cytotoxicity
Mini Stix ligature ties non-coated (TP Orthodontics)	Hernandez-Gomora et al. 2017 [39]	N/A	In Vitro	Growth inhibition of *S.mutans*, *L.casei*, *S.aureus,* and *E.coli*	Increased strength, tension and displacement	N/A	N/A
Polyurethan-based Elastomeric ligatures (Sani-Tie®, Dentsply Sirona)	Schubert et al. 2024 [40]	N/A	In Vitro	Decreased *S.mutans* adhesion	Increased surface-free energy	Decreased	N/A
Clear elastomeric ligatures (DD plus, USA)	Pasala et al. 2024 [41]	N/A	In Vitro	Growth inhibition of *S. mutans*	Decreased durability	N/A	N/A
Elastomeric ligatures (Dentalastic, Dentaurum, Ispringen, Germany)	Kim et al. 2012 [38]	N/A	In Vivo (human)	No significant effect	N/A	N/A	N/A
Elastomeric ligatures (TP Orthodontics)	Choi et al. 2024 [42]	NSF, EC, PEG, DCM	In Vitro	Growth inhibition of *S. mutans* and significantly lower than the uncoated control	No significant effect in tensile force	N/A	N/A
Elastomeric ligatures (TP Orthodontics)	Choi et al. 2025 [43]	NSF, EC, PEG, DCM	In Vitro	Significant reduction in total and aciduric bacteria and overall biofilm thickness and demineralization	N/A	N/A	N/A

NSF: nanosilver fluoride, EC: ethyl cellulose, PEG: polyethylene glycol 6000, DCM: dichloromethane; N/A: not reported in the primary literature.

**Table 6:** The properties of orthodontic acrylic (PMMA) treated with silver nanoparticles (AgNPs).

Reference	Tested Conc. of AgNPs (w/w)	Combinatory Material	Type of Study	Antibacterial Effect	Side Effect			
					Cytotoxicity	Discoloration	Tensile Strength	Surface Roughness
Ghaffari et al. 2015 [58]	5%	N/A	In Vitro	N/A	N/A	N/A	Significant decrease	N/A
Williams et al. 2017 [57]	0.4%	benzoate	In Vitro	Short term washout: antibacterial effect against *S. mutans*; Long term washout: no effect	N/A	N/A	N/A	no statistically significant difference
Ghorbanzadeh et al. 2015 [55]	0.5%	N/A	In Vivo (human)	Reduced growth and biofilm formation of *S. mutans, S. sobrinus, L. acidophilus,* and *L. casei.*: NanoAg-IS-PMMA > NanoAg-I-PMMA	N/A	N/A	N/A	N/A
Farhadian et al. 2016 [56]	500 ppm	N/A	In Vivo (human)	Antibacterial effect against *S. mutans*	No effect	No effect	N/A	N/A

PMMA: Polymethyl-methacrylate; NanoAg-I-PMMA: PMMA incorporated with silver nanoparticles; NanoAg-IS-PMMA: silver nanoparticles in situ in PMMA; N/A: not reported in the primary literature.

## Data Availability

No new data were created or analyzed in this study. Data sharing is not applicable to this article.
